# How Do Alien Plants Fit in the Space-Phylogeny Matrix?

**DOI:** 10.1371/journal.pone.0123238

**Published:** 2015-04-20

**Authors:** Şerban Procheş, Félix Forest, Sarah Jose, Michela De Dominicis, Syd Ramdhani, Timothy Wiggill

**Affiliations:** 1 Discipline of Geography, University of KwaZulu-Natal, Westville Campus, Private Bag X54001, Durban, South Africa; 2 Jodrell Laboratory, Royal Botanic Gardens, Kew, Richmond, Surrey, TW9 3DS, United Kingdom; 3 School of Life Sciences, University of KwaZulu-Natal, Westville Campus, Private Bag X54001, Durban, South Africa; WSL Institute for Snow and Avalanche Research SLF, SWITZERLAND

## Abstract

Recent advances in the field of plant community phylogenetics and invasion phylogenetics are mostly based on plot-level data, which do not take into consideration the spatial arrangement of individual plants within the plot. Here we use within-plot plant coordinates to investigate the link between the physical distance separating plants, and their phylogenetic relatedness. We look at two vegetation types (forest and grassland, similar in species richness and in the proportion of alien invasive plants) in subtropical coastal KwaZulu-Natal, South Africa. The relationship between phylogenetic distance and physical distance is weak in grassland (characterised by higher plant densities and low phylogenetic diversity), and varies substantially in forest vegetation (variable plant density, higher phylogenetic diversity). There is no significant relationship between the proportion of alien plants in the plots and the strength of the physical-phylogenetic distance relationship, suggesting that alien plants are well integrated in the local spatial-phylogenetic landscape.

## Introduction

Recently, there has been a great deal of interest in plant community phylogenetics [1], and especially in its main invasion biology projection, Darwin’s naturalization hypothesis. This hypothesis states that plants that have close indigenous relatives in a region are less likely to become naturalized, compared to those that do not [2,3,4,5,6]. The assumption behind this hypothesis is that common ancestry implies similar traits, potentially resulting in competitive exclusion, and this assumption has been recently both qualified and quantified with the introduction and measurement of the concept of phylogenetic conservatism [7,8].

While phylogenetics adds to Darwin’s original idea by converting relatedness from a categorical variable (alien plant belongs to the same genus, or not, as their closest indigenous relative) into a continuous one (the phylogenetic distance between the two), to our knowledge, no attempt has been made to convert co-occurrence into a continuous variable, although the physical (spatial) distance between plants seems to be an obvious choice. While much work in this field was performed at large scales (tens to thousands of kilometers [9,10]), the scale which is relevant for Darwin’s naturalization hypothesis is likely to be fine (phytosociological plot size or finer). Some of the most convincing results in community phylogenetics are indeed considering very small plots [11]. At large scale, alien species need to be somewhat similar to native species to share the same physical environment. Therefore, at this scale, it is likely that alien species are more related to native species than expected by random [1], thus the expectation would be a positive relationship between physical and phylogenetic distance. In the very close neighborhood, however, species interact by competing for resources. Here, to coexist, species need to be somewhat dissimilar, and it can be expected that alien species are less related to native species. It can thus be expected that alien plants would show an increased phylogenetic distance to plants in their neighborhoods, compared to native plants [3]. If so, this should result in a negative relationship between physical and phylogenetic distance.

In several previous studies, plant stems have been fully mapped for vegetation plots [12,13,14], and in at least one such case [15] the relatedness between plants has been taken into consideration. Nevertheless, this article is a first attempt—a simple and static one—at understanding how the relationship between phylogenetic distance and spatial distance is or is not affected by whether a plant is or not indigenous to the study region. Given the broad range of phylogenetic diversity values in plant communities [16], we considered it important to also include some cross-vegetational variation. In grasslands, many species co-occur in the same stratum, while forests have several layers (e.g. understorey, canopy), which could impact on the nature and intensity of plant interactions [12,14]. Furthermore, there may be differences between these two vegetation types in total plant abundance. Furthermore, since grass species (Poaceae, dominant in grassland) were the example used to illustrate Darwin’s naturalization hypothesis in the study that was most successful in this direction yet [6], we were also interested in determining whether community phylogenetic patterns are particularly strong in vegetation dominated by grasses.

## Methods

### Study area

The study was conducted in the Palmiet Nature Reserve (managed by the eThekwini/Durban Municipality) and on the neighboring Westville Campus of the University of KwaZulu-Natal in Durban, South Africa—an area naturally covered by a mosaic of grassy and woody vegetation, but having been largely engulfed by urban development during the twentieth century. The patchy nature of the remaining natural vegetation is in itself not fundamentally distinct from the natural patchiness of the two vegetation types, but both have recently become invaded by alien shrubs, vines, and small trees [17]. The borderline between forest and grassland in the region was naturally kept sharp by fire (mostly managed by campus and conservation authorities), but is now locally blurred by invasive plants [18]. Ten alien plant species are targeted by control operations in the area, given their current or potential negative impact on indigenous plant assemblages and/or human activities in the area (*Cardiospermum grandiflorum*, *Chromolaena odorata*, *Ipomoea purpurea*, *Lantana camara*, *Litsea glutinosa*, *Melia azedarach*, *Pennisetum purpureum*, *Ricinus communis*, *Solanum mauritianum*, *Tithonia diversifolia*) [17].

### Sampling

Twenty 5 x 5 m plots were selected, ten dominated by woody plants (‘forest’) and ten dominated by herbaceous plants (‘grassland’); intermediate vegetation was avoided. Considering that much of the study area is extremely steep (and including several sheer cliff faces), plots had to be selected to approximate a random distribution within the more accessible parts of the relevant vegetation patches (slope of less than 30°; see plot coordinates in S1 Table). While we noticed alien plant control teams working in neighboring areas on various occasions, we could detect no evidence of these measures having been applied in any of our plots. All plants with living above-ground parts were manually mapped in the plots by laying down a grid of string across the plot and assigning x and y values for each individual plant. This approach could result in potential errors of 5 cm or less. All data collection and mapping were performed from April to July 2008. All data points were digitized using the ArcGIS software [19], thus recording the x-y coordinates for each plant. Hawth’s tools (Geospatial Modelling Environment; www.spatialecology.com) were used to calculate the physical distance between every two plant individuals. Plants were identified to species level (either in the field or based on photographs), and their regional status (indigenous, alien) was recorded using relevant references [20,21,22]. As no plant collections were performed at the study sites, no permits were required.

### Phylogenetic diversity

Phylogenetic distances between taxa were calculated using the *phydist* option in Phylocom (4.0.1b [23]). The phylogenetic tree was reconstructed using DNA sequences from the plastid *rbcL* exon (coding for the ribulose-1,5-bisphosphate carboxylase/oxygenase large subunit) for one exemplar species for each genus found in the plots. We chose to use the *rbcL* exon as it is a widely use marker in plant phylogenetics, with very good coverage at the genus level in publicly available sequence databases, and it also presents an appropriate level of variability for the reconstruction of phylogenetic relationships across the angiosperms, as needed here [24]. DNA sequences we obtained from Genbank. The phylogenetic tree was reconstructed using the parsimony criteria as implemented in PAUP [25] (heuristic search, 1000 replicates of random addition sequence, and tree bisection reconnection branch swapping). Branch lengths were made ultrametric using the penalized likelihood approach as implemented in the software r8s [26] (see resulting tree in S1 Fig). Phylogenetic diversity measures were obtained using the same approach as described in [27], by adding branch lengths (as measured in millions of years). Using a randomization procedure (with 10,000 replicates; see [17] for details), each plot was evaluated to determine if its phylogenetic diversity is higher (overdispersed) or lower (clustered) than expected from their taxon richness.

### Statistical analyses

First, we compared the two vegetation types in terms of number of species, phylogenetic diversity, and proportional representation of alien plants using one-way ANOVA. We then correlated phylogenetic distance and physical distance by assigning the phylogenetic distance between two species to every pair of individual plants from those two species co-occurring within one plot. Thus, in most cases, multiple physical distance values corresponded to one phylogenetic distance value. We performed a Mantel tests for each plot, calculating Pearson’s correlation coefficient with 999 permutations [28,29]. We then explored how these coefficients varied between grassland and forest vegetation, and in relation to the number of species, phylogenetic diversity, plant density, and log-transformed percentage alien plants (generalized linear models, Gaussian distribution). All analyses were performed in R 2.10.0 [30]; with the mantel function from the package vegan 1.17–2 [31].

## Results

### The diversity and abundance of indigenous and alien plants

A total of 4,276 individual plants were recorded, of which 261 were aliens. These comprised 194 species assigned to 135 genera and 52 families. Alien individuals belonged to 18 species assigned to 17 genera and nine families. Only one genus (*Indigofera*) contained both indigenous and alien species (not in the same plot). The majority of the most abundant indigenous plants, in both forest and grassland, were graminoids (Poaceae and Cyperaceae), although these belonged to different species in the two vegetation types. The most abundant aliens (predominantly Asteraceae), in contract, crossed vegetation boundaries (Table 1). The high turnover in indigenous trees meant that none of these were particularly abundant overall (see S1 Table).

**Table 1:** Summary of the most abundant indigenous and alien species recorded.

Species	Total abundance	Forest plots present	Grassland plots present
Indigenous			
*Cymbopogon plurinodis* (Poaceae)	491		6
*Digitaria eriantha* (Poaceae)	395		6
*Oplismenus hirtellus* (Poaceae)	251	8	
*Cyperus albostriatus* (Cyperaceae)	249	6	
*Gerbera piloselloides* (Asteraceae)	189		9
Alien			
*Chromolaena odorata* (Asteraceae)	88	9	3
*Sonchus oleraceus* (Asteraceae)	43		7
*Sorghum halepense* (Poaceae)	27	2	
*Tagetes minuta* (Asteraceae)	25		1
*Melia azedarach* (Meliaceae)	17	1	5

### Forest-grassland comparisons

**Fig 1:**
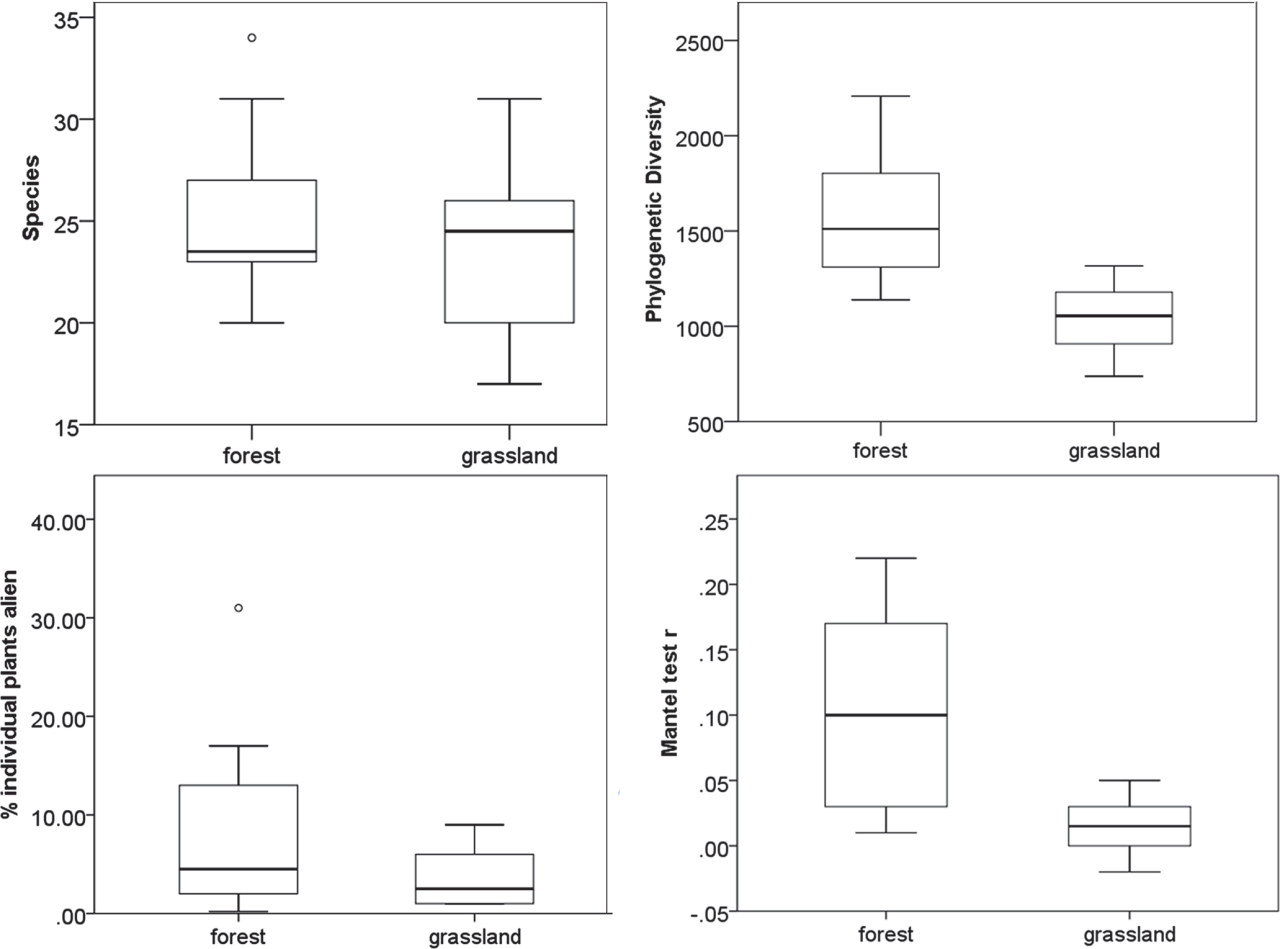
Forest-grassland comparisons. The two vegetation types are compared in terms of number of plant species, phylogenetic diversity, representation of alien species, and Mantel test r values (box-and-whisker plots).

Phylogenetic diversity values range from 1341 Ma to 2321 Ma for forest plots and from 925 Ma to 1528 Ma for grassland plots. There was no difference between the two vegetation types in terms of species numbers per plot (F = 2.220, P>0.1), but phylogenetic diversity was higher in forest (F = 18.969, P<0.0001); plant density was higher in grassland (F = 14.802, P = 0.001), and there was no difference in the percentage of plants belonging to alien species (F = 2.424, P>0.1) (Fig 1). The randomization procedure identified eight of the ten grasslands plots as clustered. None of the forest plots was clustered, but one of them had higher phylogenetic diversity than expected by chance (over-dispersed). Removing alien species resulted in virtually the same results (one additional grassland plot became clustered), whereas removing grasses had a more substantial effect—in this case several of the otherwise clustered grassland plots were no longer clustered (but four such plots remained), and one forest plot became clustered.

### Mantel tests

**Fig 2:**
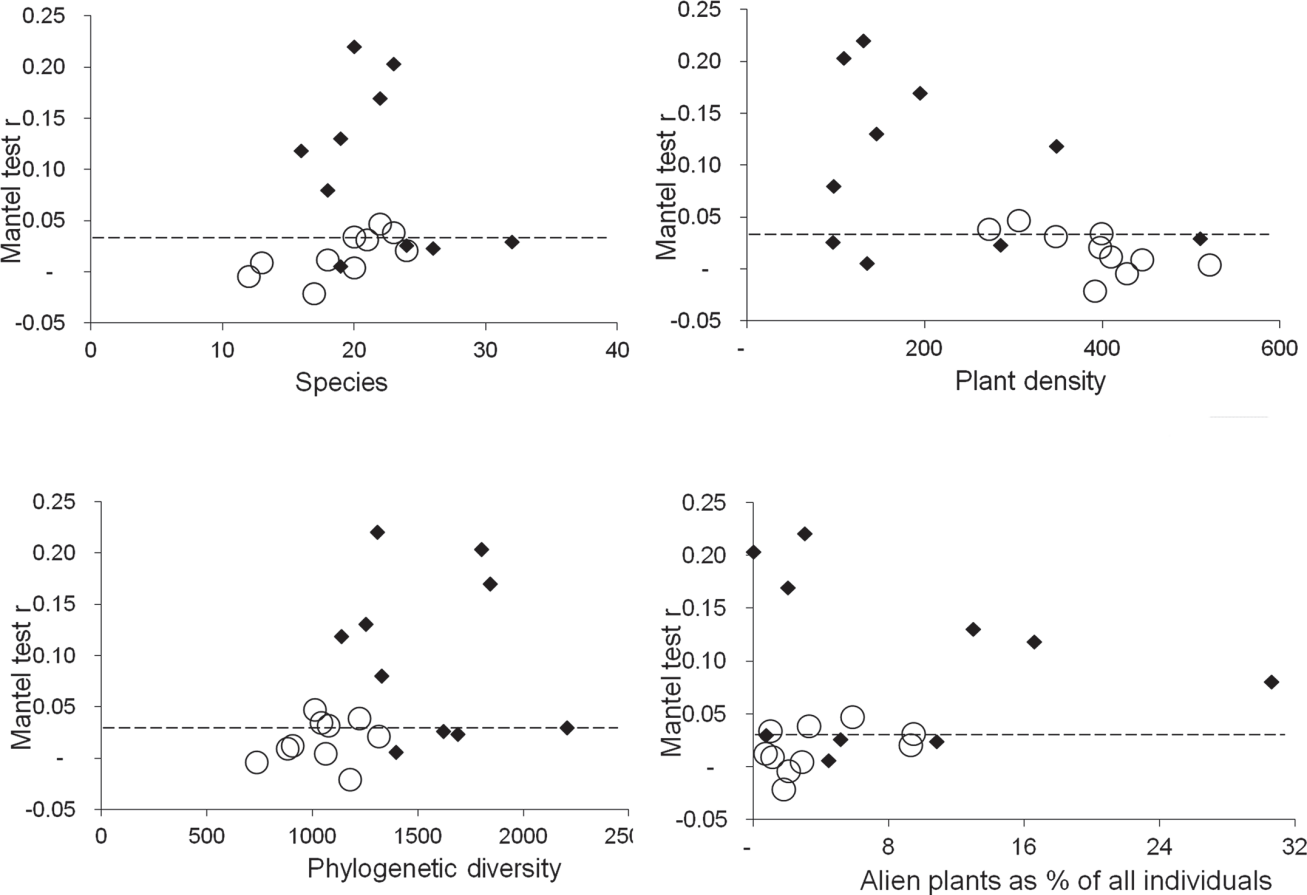
Factors potentially influencing the results on Mantel tests in our plots. Graphs exploring links between different plot-level variables and Mantel test r values for grassland (circles) and forest (diamonds) plots. Points above the dashed line indicate significant Mantel tests.

Mantel test r values were mostly positive (always positive and mostly significant in forest, mostly positive and seldom significant in grassland; Fig 2)—overall significantly lower in grassland (F = 10.709; P = 0.004; Fig 1). When using generalised linear models to express Mantel test r values as a function of vegetation type and other variables, the only significant effect was that of vegetation type on the way in which species richness influenced the value of Mantel’s r (t = -2.155, P = 0.0467). The effect of the percentage of alien plants in the assemblage on Mantel’s r was not significant (t = -1.867, P = 0.0816; this was however the second lowest of the twelve P values across all the generalised linear models—including interactions). On the two-dimensional graphs visually exploring links between different plot-level variables and Mantel test r values (Fig 2), grassland sites formed separate clusters from the bulk of the forest sites, but in most cases there were a few forest plots overlapping the grassland cluster. These patterns were in no obvious way linked to those of phylogenetic clustering or over-dispersion described above. The only clear separation between grassland and forest plots was in the case of phylogenetic diversity. Although grassland and forest values overlapped in both phylogenetic diversity and Mantel r values, grassland sites formed a fairly distinct cluster in the two-dimensional graph, with low values in both (Fig 2).

## Discussion

### Potential caveats

Although we used a genus-level phylogenetic tree, this is very unlikely to have affected our results substantially, as congeneric species very seldom occurred in the same plot—and this was never the case with indigenous and alien congenerics. Far more important in taking our results further could be recording plant sizes, and even more so, following changes in plant size and survival in plots through time. Once time is taken into consideration, the importance of the indigenous/alien status may emerge. Woody invaders often reduce the survival of other seedlings [32]. This is not necessarily linked to how closely related they are [33], but interactions between phylogenetic distance and plant status in the region are to be expected anyway [34]. Furthermore, an assessment of the specific types of competitive interactions (“phenotypic matching” versus “phenotypic differences”; [5]) will probably add further depth to studies of this kind once these are better understood.

Peculiarities of the system studied here, such as the remarkable patchiness of vegetation types (both natural and anthropogenic) and the high turnover of indigenous tree species in forest, could mean that our results should not be directly extrapolated elsewhere. Nevertheless, several points are remarkable and probably not unique to the system we studied.

### Birds of a feather flock together

In our study, only positive relationships between physical distance and phylogenetic distance were significant (plants that are closely related occur closer to each other), but this was less likely in plots with denser vegetation and where graminoid plants were dominant (Fig 2). The absence of significant negative relationships is remarkable, considering that our method should allow picking up interactions across finer scales than elsewhere [34].

Grassland plots in general had fewer significant relationships than forest plots, but this may only indicate that, in this case, the tendency of related plants to track similar resources is compensated by competitive interactions. Darwin’s naturalisation hypothesis is supported when considering grasses alone [9], which is less likely to be shown in other plants [35]. Furthermore, grasses are among several families locally overrepresented on invasive species lists, which can result in a phylogenetic clustering effect for the vegetation as a whole [36]. The forest plots more similar to grassland in terms of Mantel r values either had lower levels of invasion (similar to grassland) or actually contained high numbers of grass or sedge individuals. Grassy systems as a whole have been shown to represent clustered subsets of the angiosperm assemblages present regionally [16], although within grasslands there is no clear link between niche and phylogenetics [37]. This can explain why grassland appears clustered when the random model is a grassland-forest species mix, as analysed here, but without significant Mantel test results.

The significant effect of vegetation type on the species richness-Mantel’s r relationship can be described as follows: the grassland plots, as well as those forest plots that are species rich, show low values for Matel’s r. Conversely, forest plots that are species poor (and have lower understorey cover) have high values for Mantel’s r. The high tree diversity in our study area (even in plots with overall low species diversity) means that our results are complementary to the finding of competition though spatial point-patterns analysis in species-poor Boreal forests [14]. The interactions in the herbaceous layer are complex, and the effects of competition and positive interactions [12] may cancel out and result in non-significant Mantel tests.

### Aliens behave local

Most importantly for the central question of the study, we did not find a significant effect of the level of invasion (measured as the proportion of plants belonging to alien species) on the physical distance-phylogenetic distance relationship. This is surprising given the dynamic nature of the invasion in the studied system [38], but fits in with the lack of significant difference between indigenous and alien plants as regards a variety of morphological traits [39]. Nevertheless, given the fact that the P value was in this case close to significance level, we cannot exclude the possibility that such effect will be detected in future studies, particularly if conducted in vegetation with different properties. In particular, a broader range of invasion level values, and species-poor tree-dominated assemblages could hold promise in detecting significant effects.

## Conclusion

Our results indicate that alien plants are, at least in the systems studied here, by-and-large fitting well in the indigenous phylogenetic landscape—although this picture may change with the stages of invasion [40,41]. This could even be the case in our study area, given the rapid invasion dynamics documented at our sites [38].

Methodologically, we show that analyses measuring physical distances between plants can complement plot-level analyses that are currently widely used. In particular, these should be employed in longitudinal long-term studies with competition in mind—in which case they would provide a complex tool, especially relevant in invasion ecology.

## Supporting Information

S1 Fig The phylogenetic tree produced for the study. (DOCX)

S1 Table The representation of alien and indigenous plant species across forest and grassland plots as analysed in the study. (XLS)
