# Supplementary material for: How Do Alien Plants Fit in the Space-Phylogeny Matrix?
Source: PLoS One. 2015 Apr 20;10(4):e0123238. doi: 10.1371/journal.pone.0123238 (PMC4403803; doi:10.1371/journal.pone.0123238)
Supplement: S1 Fig — (DOCX) [file pone.0123238.s001.docx]

**
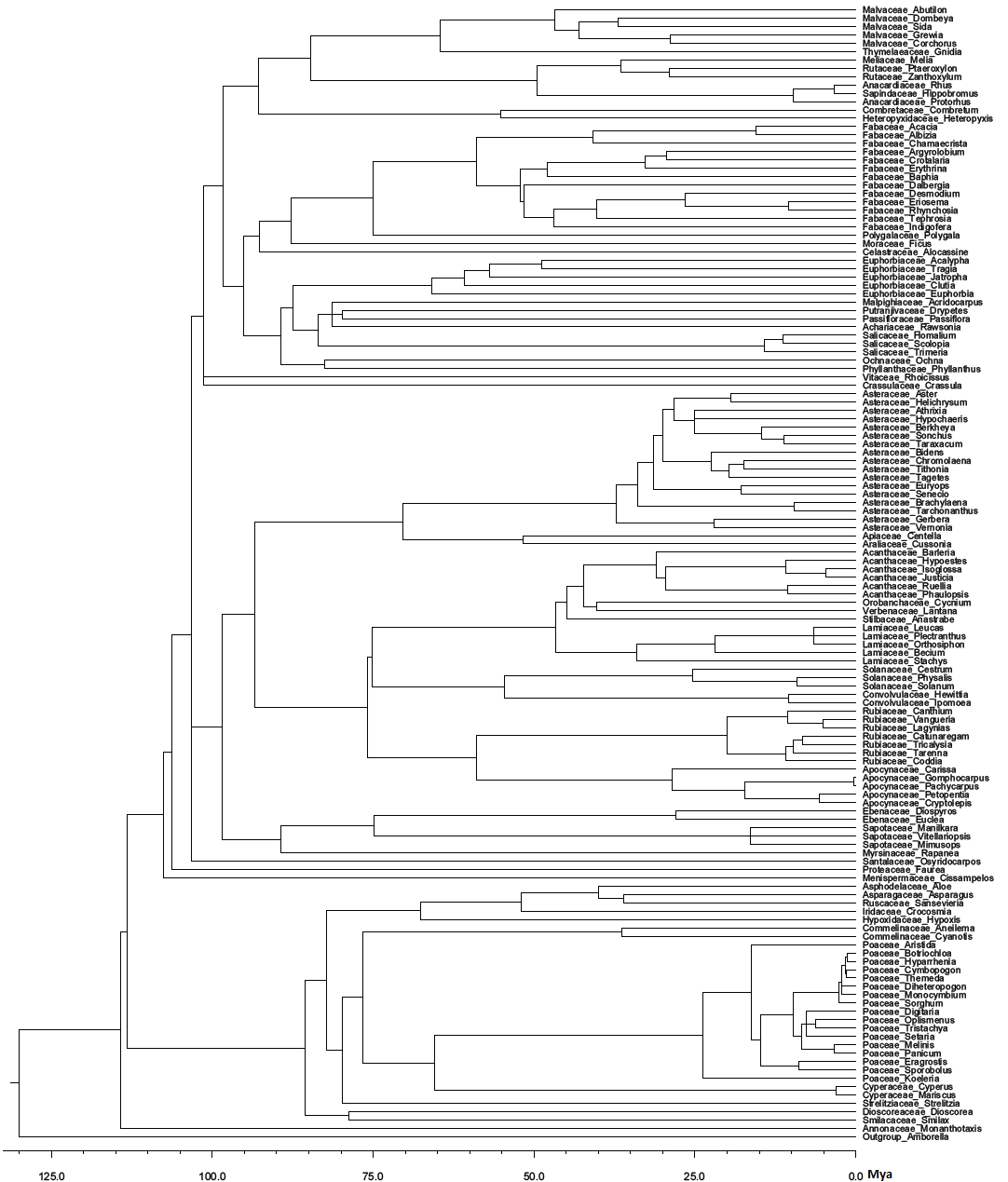
**

**S1 Figure. The phylogenetic tree produced for the study.** Full Newick-format tree: ((((((((((((Malvaceae_Abutilon:46.811895,((Malvaceae_Dombeya:36.961663,Malvaceae_Sida:36.961663):6.117979,(Malvaceae_Grewia:28.833867,Malvaceae_Corchorus:28.833867):14.245775):3.732253):17.837621,Thymelaeaceae_Gnidia:64.649516):20.039943,((Meliaceae_Melia:36.52245,(Rutaceae_Ptaeroxylon:29.027983,Rutaceae_Zanthoxylum:29.027983):7.494467):13.048471,((Anacardiaceae_Rhus:3.29971,Sapindaceae_Hippobromus:3.29971):6.419011,Anacardiaceae_Protorhus:9.71872):39.8522):35.118539):8.096909,(Combretaceae_Combretum:55.140755,Heteropyxidaceae_Heteropyxis:55.140755):37.645613):5.551707,(((((((Fabaceae_Acacia:15.478264,Fabaceae_Albizia:15.478264):25.414345,Fabaceae_Chamaecrista:40.892608):18.026512,((((Fabaceae_Argyrolobium:29.41821,Fabaceae_Crotalaria:29.41821):3.26518,Fabaceae_Erythrina:32.68339):15.22444,Fabaceae_Baphia:47.90783):4.181573,(Fabaceae_Dalbergia:51.532348,(((Fabaceae_Desmodium:26.546796,(Fabaceae_Eriosema:10.375072,Fabaceae_Rhynchosia:10.375072):16.171725):13.699892,Fabaceae_Tephrosia:40.246688):6.656833,Fabaceae_Indigofera:46.903521):4.628827):0.557055):6.829717):16.084001,Polygalaceae_Polygala:75.003121):12.718416,Moraceae_Ficus:87.721537):5.005805,Celastraceae_Alocassine:92.727342):2.459128,((((((Euphorbiaceae_Acalypha:48.856279,Euphorbiaceae_Tragia:48.856279):8.076205,Euphorbiaceae_Jatropha:56.932483):3.874412,Euphorbiaceae_Clutia:60.806896):5.084081,Euphorbiaceae_Euphorbia:65.890977):21.495613,((Malpighiaceae_Acridocarpus:81.305915,(Putranjivaceae_Drypetes:79.764615,Passifloraceae_Passiflora:79.764615):1.5413,Achariaceae_Rawsonia:81.305915):2.302468,((Salicaceae_Homalium:11.316169,Salicaceae_Scolopia:11.316169):2.822388,Salicaceae_Trimeria:14.138557):69.469826):3.778206):1.913411,(Ochnaceae_Ochna:82.494795,Phyllanthaceae_Phyllanthus:82.494795):6.805205):5.886469):3.151606):3.080429,Vitaceae_Rhoicissus:101.418504,Crassulaceae_Crassula:101.418504):1.777835,(((((((((Asteraceae_Aster:19.396056,Asteraceae_Helichrysum:19.396056):8.898652,(Asteraceae_Athrixia:25.062691,Asteraceae_Hypochaeris:25.062691,(Asteraceae_Berkheya:14.617109,(Asteraceae_Sonchus:11.222873,Asteraceae_Taraxacum:11.222873):3.394236):10.445581):3.232017):1.648542,(Asteraceae_Bidens:22.412313,((Asteraceae_Chromolaena:17.378635,Asteraceae_Tithonia:17.378635):2.25794,Asteraceae_Tagetes:19.636574):2.775739):7.530938):1.437711,(Asteraceae_Euryops:17.869763,Asteraceae_Senecio:17.869763):13.511198):2.46184,(Asteraceae_Brachylaena:9.568132,Asteraceae_Tarchonanthus:9.568132):24.27467):3.326554,(Asteraceae_Gerbera:21.999169,Asteraceae_Vernonia:21.999169):15.170187):33.255146,(Apiaceae_Centella:51.659726,Araliaceae_Cussonia:51.659726):18.764775):23.015793,((((((Acanthaceae_Barleria:30.971176,((Acanthaceae_Hypoestes:10.884485,(Acanthaceae_Isoglossa:4.59774,Acanthaceae_Justicia:4.59774):6.286745):18.684237,(Acanthaceae_Ruellia:10.653817,Acanthaceae_Phaulopsis:10.653817):18.914905):1.402454):11.29405,(Orobanchaceae_Cycnium:40.296191,Verbenaceae_Lantana:40.296191):1.969034):2.584846,Stilbaceae_Anastrabe:44.850071):1.784666,(((Lamiaceae_Leucas:6.58294,Lamiaceae_Plectranthus:6.58294,Lamiaceae_Orthosiphon:6.58294):15.361381,Lamiaceae_Becium:21.944321):12.153024,Lamiaceae_Stachys:34.097345):12.537393):28.586823,((Solanaceae_Cestrum:25.290543,(Solanaceae_Physalis:9.148079,Solanaceae_Solanum:9.148079):16.142464):29.320649,(Convolvulaceae_Hewittia:10.477742,Convolvulaceae_Ipomoea:10.477742):44.13345):20.610368):0.599534,(((Rubiaceae_Canthium:10.634049,(Rubiaceae_Vangueria:5.05276,Rubiaceae_Lagynias:5.05276):5.581289):9.356456,(((Rubiaceae_Catunaregam:8.236537,Rubiaceae_Tricalysia:8.236537):1.541459,Rubiaceae_Tarenna:9.777996):1.11836,Rubiaceae_Coddia:10.896357):9.094148):38.971687,(Apocynaceae_Carissa:28.553238,((Apocynaceae_Gomphocarpus:0.350303,Apocynaceae_Pachycarpus:0.350303):16.887041,(Apocynaceae_Petopentia:5.690072,Apocynaceae_Cryptolepis:5.690072):11.547272):11.315894):30.408953):16.858903):17.6192):5.050845,(((Ebenaceae_Diospyros:28.004037,Ebenaceae_Euclea:28.004037):46.907032,(Sapotaceae_Manilkara:16.311726,Sapotaceae_Vitellariopsis:16.311726,Sapotaceae_Mimusops:16.311726):58.599343):14.38893,Myrsinaceae_Rapanea:89.3):9.19114):4.7052,Santalaceae_Osyridocarpos:103.196339):3.15017,Proteaceae_Faurea:106.34651):1.233227,Menispermaceae_Cissampelos:107.579736):5.682169,(((((Asphodelaceae_Aloe:40.024539,(Asparagaceae_Asparagus:36.066393,Ruscaceae_Sansevieria:36.066393):3.958146):11.904031,Iridaceae_Crocosmia:51.92857):15.687189,Hypoxidaceae_Hypoxis:67.615759):14.659658,(((Commelinaceae_Aneilema:36.382604,Commelinaceae_Cyanotis:36.382604):40.25366,(((Poaceae_Aristida:16.229045,((((((Poaceae_Botriochloa:1.296518,Poaceae_Hyparrhenia:1.296518):0.286853,(Poaceae_Cymbopogon:1.427302,Poaceae_Themeda:1.427302):0.156068):0.555529,Poaceae_Diheteropogon:2.1389,Poaceae_Monocymbium:2.1389):0.42793,Poaceae_Sorghum:2.56683):7.215535,((Poaceae_Digitaria:7.679598,(Poaceae_Oplismenus:6.181728,Poaceae_Tristachya:6.181728):1.49787,Poaceae_Setaria:7.679598):0.750437,(Poaceae_Melinis:3.379454,Poaceae_Panicum:3.379454):5.050581):1.35233):4.982969,(Poaceae_Eragrostis:8.879564,Poaceae_Sporobolus:8.879564):5.88577):1.463711):7.570375,Poaceae_Koeleria:23.79942):41.70058,(Cyperaceae_Cyperus:3.101658,Cyperaceae_Mariscus:3.101658):62.398342):11.136263):3.174679,Strelitziaceae_Strelitzia:79.810942):2.464475):3.324335,(Dioscoreaceae_Dioscorea:78.714426,Smilacaceae_Smilax:78.714426):6.885326):27.662154):1.025628,Annonaceae_Monanthotaxis:114.287533):15.712467,Outgroup_Amborella:130.0);
